# A Novel Sesquiterpene from *Callistephus chinensis* Improves Alcohol-Induced Liver Disease by Regulating the AMPK/NF-κB Signaling Pathway and Gut Flora

**DOI:** 10.3390/molecules30224371

**Published:** 2025-11-12

**Authors:** Bingxin Zhang, Ning Wang, Xiaoxu Chen, Nan Yang, Ying Zhao, Xiaoshu Zhang

**Affiliations:** School of Functional Food and Wine, Shenyang Pharmaceutical University, Shenyang 110016, China; 13177089059@163.com (B.Z.); 15094062128@163.com (N.W.); cxx18456199339@163.com (X.C.); yangn231026@163.com (N.Y.)

**Keywords:** Callistephus A, anti-inflammatory, hepatic steatosis, gut flora, short-chain fatty acids, transcriptome analysis

## Abstract

Alcoholic liver disease (ALD) caused by excessive alcohol consumption poses a serious threat to human health. *Callistephus chinensis* (L.) Nees is an herb of the Asteraceae family that has good results in the prevention and treatment of a variety of liver diseases, including multifactorial liver injury, non-alcoholic fatty liver disease/non-alcoholic steatohepatitis, liver fibrosis. Sesquiterpenes are thought to be biologically active components that typically have anti-inflammatory, immunomodulatory, and cardioprotective effects. Callistephus A (CA) is a sesquiterpene compound with a rare 6/7 ring skeleton, which has been isolated only from the *Callistephus chinensis* Nees. However, the mechanism of action of CA on alcoholic liver disease remains unclear. In this study, alcoholic liver mice were fed with 20 and 40 mg/kg CA, respectively, for 11 days. This study showed that CA improved hepatic steatosis and oxidative stress associated with alcohol consumption. CA alleviated liver inflammation by inhibiting the TLR4/MYD88/NF-κB pathway, ameliorating gut imbalance by restoring the abundance of *Akkermansia*, and restoring short-chain fatty acids in the gut. Transcriptome analysis revealed that CA primarily affects genes involved in lipid metabolism and inflammation. In vitro, by adding inhibitors of TLR4 (TAK-242) and AMPK (Dorsomorphin), it was confirmed that CA alleviates ALD by inhibiting TLR4 and activating AMPK. This study is the first to demonstrate that CA protects against alcoholic liver disease through the regulation of the gut flora and modulation of the AMPK/NF-κB pathway. In conclusion, CA can effectively improve alcoholic liver disease and can be used as an effective candidate drug with liver-protective effects.

## 1. Introduction

Alcoholic liver disease (ALD), the most common form of chronic liver disease, is associated with prominent morbidity and mortality worldwide [1]. Alcohol can interfere with the proper functioning of several organs, including the liver and intestines. Alcohol metabolism occurs primarily in the liver [2]. Consequently, the liver is susceptible to the effects of alcohol consumption. ALD begins with simple hepatic steatosis and can progress to alcoholic steatohepatitis, fibrosis, and liver cirrhosis. Alcohol consumption can alter the gut microbiota composition even before the onset of liver disease [3]. The gut microbiota not only determine the homeostasis of the intestinal microenvironment and human health but also play an indispensable role in the development and progression of several liver diseases [4].

Adenosine monophosphate-activated protein kinase (AMPK) is a lipid-regulating kinase that is crucial for regulating the pathogenesis of alcoholic steatosis [5]. AMPK phosphorylation also inhibits the activity of sterol regulatory element binding protein 1c (SREBP-1c) in hepatocytes [6], which reduces lipid deposition. SIRT1 plays an important role in regulating lipid metabolism and inflammation. Alcohol intake reduces SIRT1 levels, causing hepatic lipid accumulation and inflammatory reactions. Inflammation is crucial in the underlying pathogenesis of ALD [7]. Toll-like receptors (TLRs) recognize pathogen-associated molecular patterns, enabling the host to detect microbial infections and [8] are associated with inflammatory liver disease [9]. Toll-like receptor 4 (TLR4), a Lipopolysaccharide (LPS) receptor, triggers the myeloid differentiation factor 88 (MYD88)-dependent signaling pathway. This pathway activates nuclear factor κB (NF-κB) and stimulates the production of proinflammatory interferon [10]. AMPK/NF-κB signaling axis disorder is thought to be closely related to ALD [11]. Activation of AMPK not only ameliorates hepatic lipid metabolism but also directly phosphorylates and inhibits NF-κB, thereby reducing the production of pro-inflammatory cytokines. Conversely, excessive alcohol consumption suppresses AMPK activity, leading to uncontrolled activation of NF-κB and persistent hepatic inflammation. Furthermore, there is currently no effective and safe drug to treat ALD, highlighting the urgent need for a solution.

**Figure 1:**
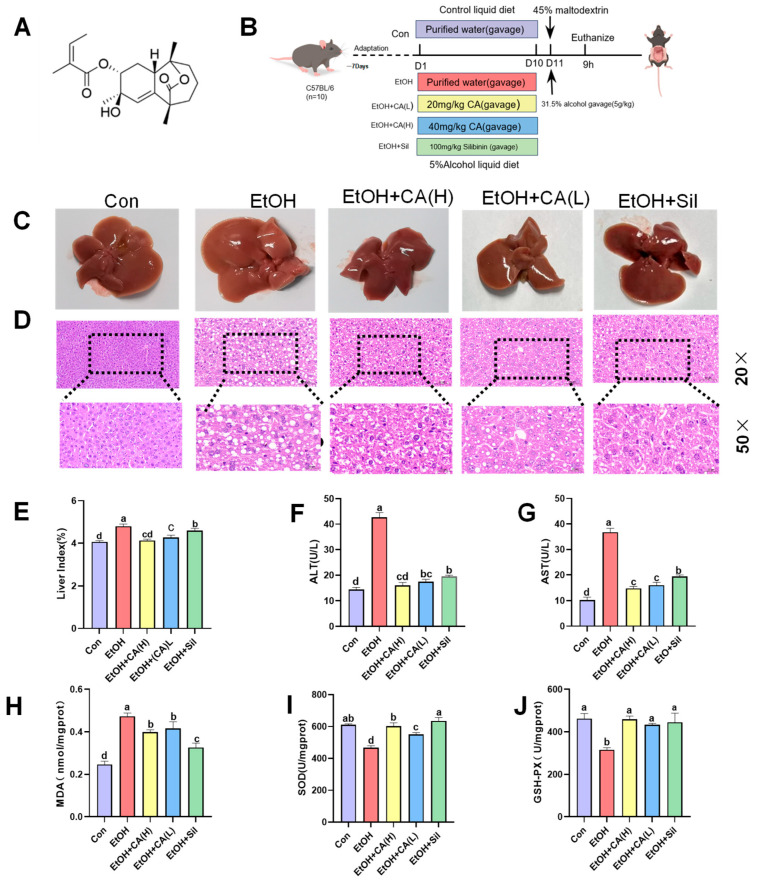
CA improved alcohol-induced liver injury. (**A**) Structure of Callistephus A. (**B**) Experiment schematic. (**C**) Gross morphologies of the livers. (**D**) H&E stained photomicrographs of a liver section (20.0× and 50.0× magnification). (**E**) Liver index (*n* = 10). (**F**) ALT and (**G**) AST serum levels (*n* = 6). (**H**) MDA, (**I**) SOD, and (**J**) GSH-Px in the liver. Data are expressed as the mean ± SD (*n* = 6). Data are expressed as mean ± standard deviations. Different letters (a–d) represent significant differences among groups (*p* < 0.05, *n* = 3), determined by one-way ANOVA using Duncan′s post-test.

The overuse of chemical drugs in the treatment of liver diseases is a major cause of drug-induced liver injury. In contrast, natural medicines have garnered significant attention due to their relatively fewer side effects and higher safety profile. Research has shown that various plant-derived active ingredients demonstrate hepatoprotective effects, effectively mitigating liver inflammation and preventing the occurrence of liver damage [12]. *Callistephus chinensis Nees* is an annual or biennial herbaceous plant belonging to the genus Chrysanthemum within the Asteraceae family. *Callistephus chinensis Nee* has been traditionally used for protecting the eyes and liver, and is thus considered a potential hepatoprotective agent for the treatment and prevention of liver-related diseases. Multiple lines of evidence indicate that this plant exhibits significant pharmacological activities, including hepatoprotective, anti-inflammatory, and antioxidant effects [13,14]. Our previous study further confirmed that the extract of *Callistephus chinensis* significantly reduced hepatic fat accumulation in mice fed a high-fat diet, which prompted us to investigate its sesquiterpenoid constituents [15,16]. Callistephus A (CA) is a sesquiterpene compound with a rare 6/7 ring skeleton, which has been isolated only from the *Callistephus chinensis* Nees. Its structure is shown in Figure 1A. Our previous study demonstrated that CA exhibits protective effects against both Con A-induced immune liver injury in mice and TGF-β1-induced LX-2 cell model. In addition, CA has been shown to affect the liver function through cellular responses to oxygenates, lipids, TGF-β signaling, and NF-κB signaling. However, the protective mechanisms of CA against ALD remain unclear. Herein, we investigated the role of CA in ameliorating ALD, providing a solid theoretical foundation for the clinical application of CA as a hepatoprotector.

## 2. Results

### 2.1. CA Ameliorated Alcohol-Induced Liver Injury

In order to understand the effect of CA on ALD, we established an animal model of alcoholic liver injury, as shown in Figure 1B. The surface morphologies of liver samples from the EtOH group compared with those from the Con group showed that alcohol stimulated inflammation on the liver surface (Figure 1C); the addition of CA ameliorated this change. To assess the extent of alcohol-induced liver injury, we performed histopathological examinations as well as liver index and serum ALT and AST activity measurements. H&E staining showed a significantly higher progression of liver injury in the EtOH group compared with the Con group (Figure 1D), indicating that EtOH feeding induced inflammatory infiltration and structural lesions in hepatocytes. CA ameliorated the inflammatory manifestations. Similarly, the liver index and the AST and ALT activities were elevated in the EtOH group compared with the Con group due to alcohol stimulation, while the EtOH+CA group exhibited significantly lower levels compared with the EtOH group (Figure 1E–G). To determine whether alcohol consumption causes lipid peroxidation in the liver, we evaluated the hepatic MDA levels, which reflected the lipid peroxidation levels. The MDA levels in the EtOH group were significantly higher than those of the Con group and were effectively reduced in the EtOH+CA group (Figure 1H). We also measured the SOD and GSH-Px index levels in the liver. Compared with the Con group, the liver tissues of the EtOH group exhibited significantly reduced SOD and GSH-Px levels, which were restored by CA (Figure 1I,J. These results showed that CA reduced alcohol-induced lipid peroxidation by regulating the antioxidant enzyme levels.

### 2.2. Effect of CA on the Liver Transcriptome

**Figure 2:**
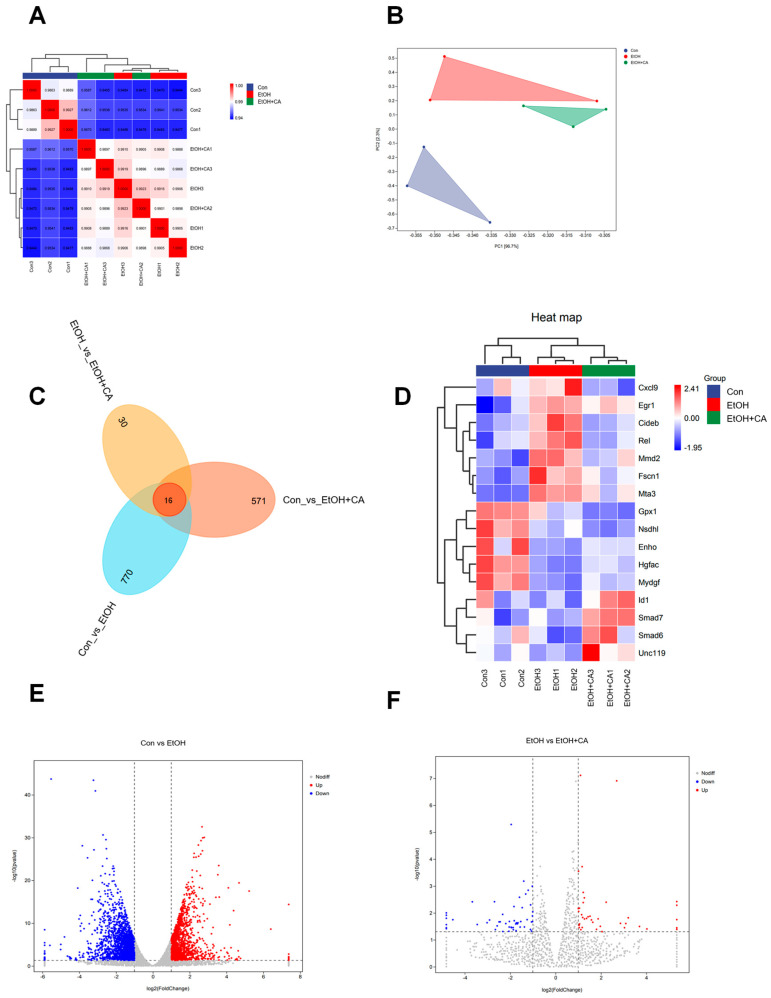
Transcriptomic effects of CA on ALD. (**A**) Pearson correlation coefficient. (**B**) Principal component analysis. (**C**) Venn diagram of DEGs. (**D**) Heatmap of the 14 shared DEGs. (**E**,**F**) Volcano plot of DEGs (*n* = 3).

To further investigate how CA ameliorates alcoholic liver injury, we performed high-throughput transcriptome analysis on liver tissues from control, EtOH-, and CA(H)-treated mice in our in vivo experiments. Pearson correlation analysis and Principal Component Analysis (PCA) demonstrated strong correlations among samples and revealed distinct separation between the CA(H) and EtOH groups (Figure 2A,B). A Venn diagram illustrated 16 common differentially expressed genes (DEGs) across comparisons (Figure 2C). The heatmap of these 16 shared DEGs showed that CA reversed the expression of some EtOH-induced DEGs (Figure 2D). Volcano plots identified 1843 downregulated and 1453 upregulated DEGs in the control group compared to the EtOH group, whereas the EtOH group showed 52 downregulated and 39 upregulated DEGs compared to the EtOH+CA(H) group (Figure 2E,F).

### 2.3. Functional Annotation and Gene Set Enrichment Analysis

**Figure 3:**
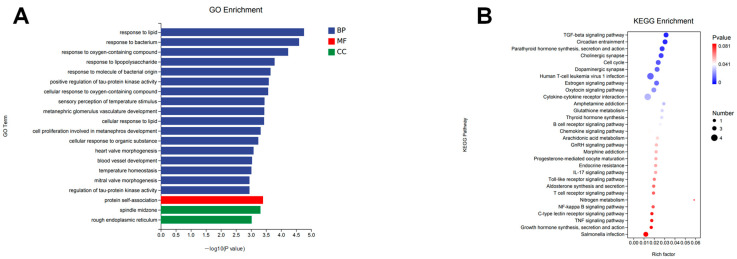
Functional annotation and gene enrichment analysis diagram of CA. (**A**) GO enrichment of DEGs. (**B**) KEGG enrichment of DEGs bubble plot (*n* = 3).

Following differential gene expression analysis, we systematically categorized genes exhibiting significant differential expression between experimental groups based on genomic annotation information. Using the differentially expressed genes (DEGs) identified from the EtOH and EtOH+CA groups, we constructed gene sets for subsequent functional enrichment analyses. Gene Ontology (GO) analysis was performed on these gene sets, covering the three main categories: biological process, cellular component, and molecular function. GO enrichment analysis results (Figure 3A) showed that DEGs were significantly enriched in various metabolic and regulatory pathways, including cellular response to oxygen-containing compounds and cellular response to lipids. KEGG results (Figure 3B) indicated involvement of the transforming growth factor β (TGF-β) signaling pathway and the Toll-like receptor (TLR) signaling pathway, further demonstrating that these differentially expressed genes are primarily associated with inflammation and lipid metabolism. Importantly, the identified GO and KEGG pathways are closely related to key pathological mechanisms of ALD, such as oxidative stress, inflammatory activation, and dysregulated lipid homeostasis. These results suggest that CA may improve alcoholic liver disease by modulating lipid accumulation and inflammation.

### 2.4. CA Improved Alcohol-Induced Hepatic Lipid Accumulation

**Figure 4:**
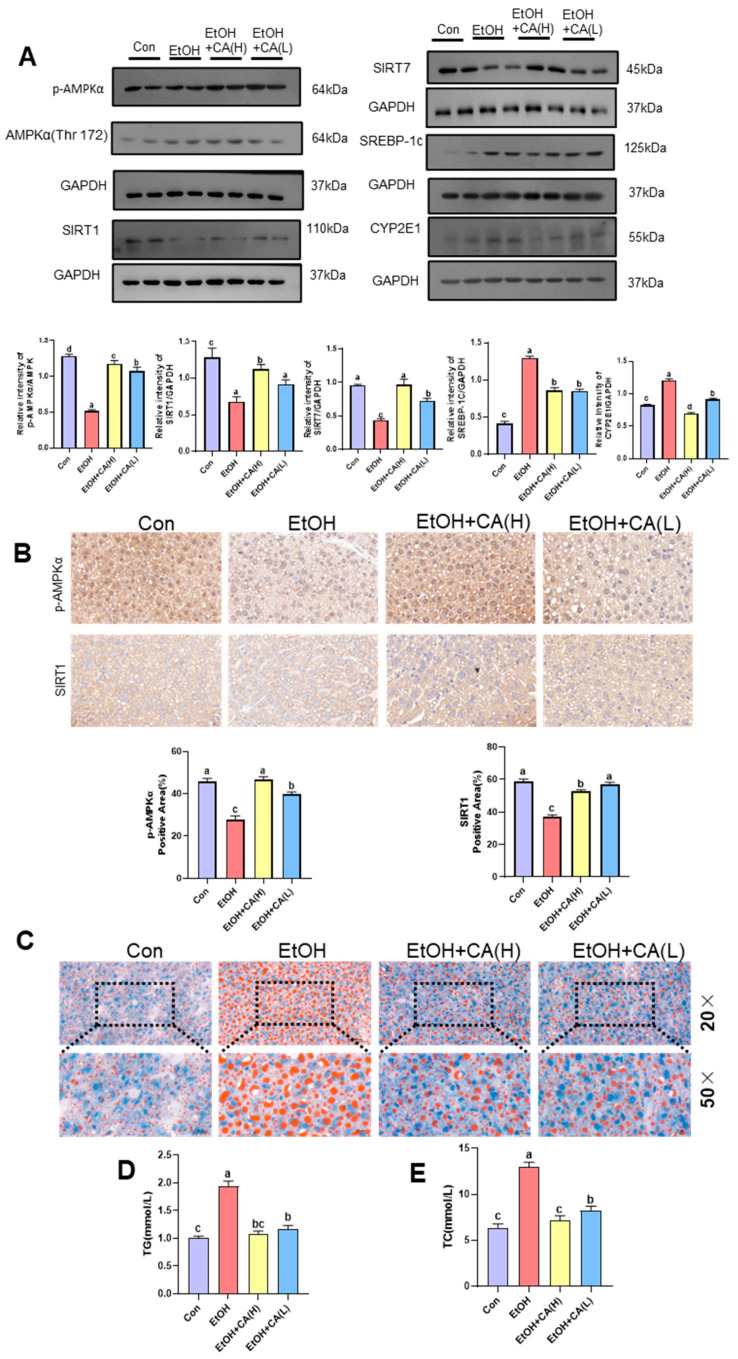
CA reduced lipid accumulation by regulating lipid synthesis and metabolism. (**A**) Hepatic expression levels p-AMPKα, AMPKα (Thr 172), SIRT1, SREBP-1c, and CYP2E1, quantified by WB (*n* = 3). (**B**) IHC staining of p-AMPKα and SIRT1 in mouse liver (50× magnification; *n* = 3). (**C**) Oil Red O stained photomicrographs of liver sections (20.0× and 50.0× magnification). (**D**) TG and (**E**) TC serum levels of TG (*n* = 6). Data are expressed as mean ± standard deviation. Different letters (a–d) represent significant differences among groups (*p* < 0.05, *n* = 3), determined by one-way ANOVA using Duncan′s post-test.

Transcriptomic analysis revealed that CA likely alleviates alcoholic liver injury by modulating lipid metabolism and ameliorating inflammation. To investigate this, we first analyzed lipid metabolism-related proteins in the livers of ALD mice using Western blot. As shown in Figure 4A, the expression levels of phosphorylated AMPK (p-AMPK), SIRT-1, and SIRT-7 were significantly decreased in the livers of the EtOH group, while the protein expression levels of SREBP-1c and CYP2E1 were markedly increased. Our results indicate that CA synergistically improves lipid metabolism by activating the AMPK/SIRT1 axis. CA treatment significantly elevated the protein levels of p-AMPK and SIRT1, suggesting the initiation of a core regulatory pathway for cellular energy metabolism. The activated AMPK, in concert with SIRT7, inhibited the key lipogenic transcription factor SREBP-1c, thereby effectively reducing hepatic lipogenesis. Concurrently, the upregulation of SIRT1 was closely associated with the downregulation of CYP2E1, indicating that CA also alleviates oxidative stress, providing a favorable microenvironment for the improvement of lipid metabolism. In summary, CA acts on multiple targets to coordinately regulate lipid synthesis and oxidative stress, ultimately mitigating hepatic lipid accumulation. As shown in Figure 4B, p-MAPK and SIRT1 exhibited consistent results with the Western blot analysis.

Oil Red O staining further detected alcohol-induced hepatic lipid infiltration (Figure 4C). Numerous red lipid droplets were observed in the hepatocytes of mice in the EtOH group. This number was significantly reduced in the liver tissue of mice in the CA group, suggesting that CA may improve alcohol-induced steatosis and reduce hepatic lipid accumulation. The serum TG and TC levels, which also reflect the lipid levels in mice, were significantly higher in the EtOH group than those in the Con group. CA reduced TG, TC, and lipid levels (Figure 4D,E).

### 2.5. CA Improved Inflammatory Response

**Figure 5:**
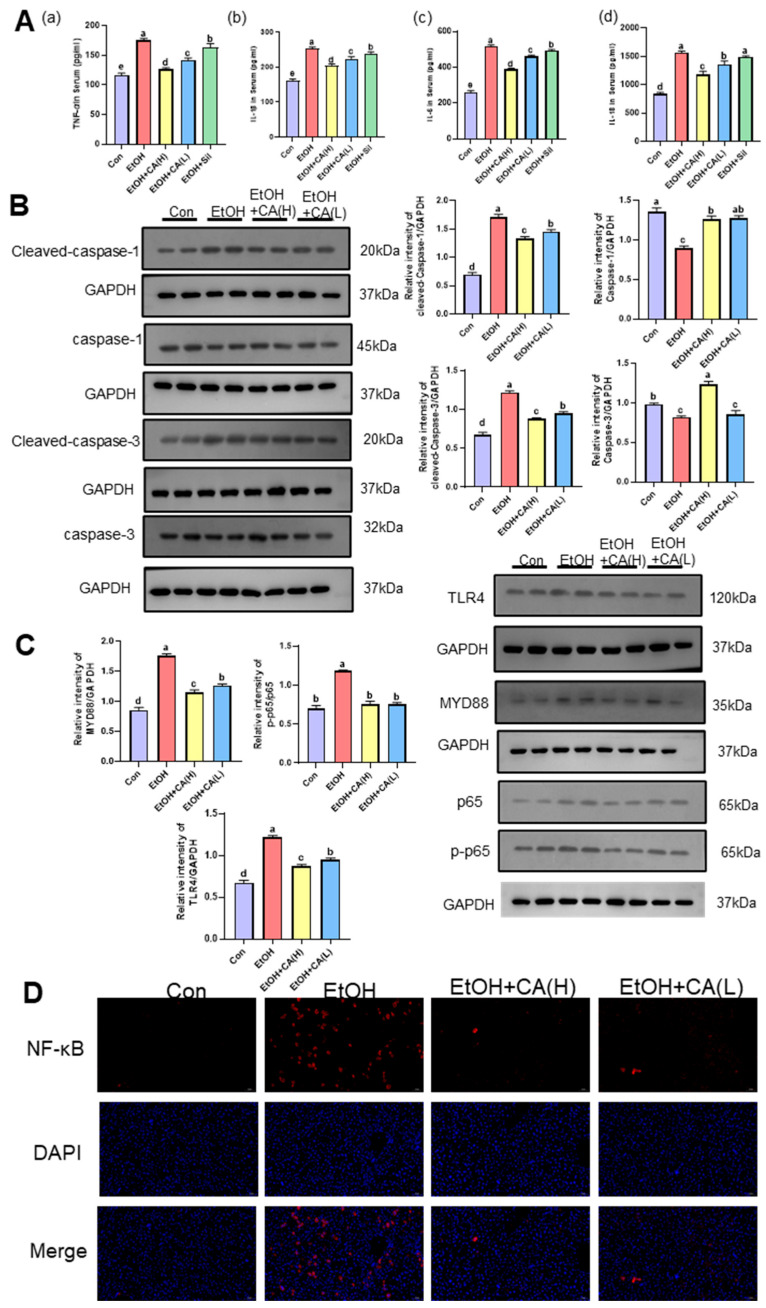
CA improved alcohol-induced liver inflammation. (**A**) Expression levels of (**a**) TNF-α, (**b**) IL-1β, (**c**) IL-6, and (**d**) IL-18, determined by ELISA. (**B**) Hepatic expression levels of Cleaved-caspase 1, caspase 1, Cleaved-caspase 3, and caspase 3, quantified by WB. (**C**) Hepatic expression levels MYD88, TLR4, NF-κB p65, and p-NF-κB p65, quantified by WB. (**D**) IF stained liver tissue samples showing the cellular localization of NF-κB p65 (50× magnification; *n* = 3). Data are expressed as the mean ± SD. Data are expressed as mean ± standard deviation. Different letters (a–e) represent significant differences among groups (*p* < 0.05, *n* = 3), determined by one-way ANOVA using Duncan′s post-test.

Transcriptomic results suggest that CA may improve alcoholic liver disease by modulating inflammation. Inflammatory cytokine levels can indicative of the inflammatory status of livers in ALD mice. Figure 5A shows that the TNF-α, IL-1β, IL-6, and IL-18 levels in the livers of mice in the EtOH group were significantly higher than those in the Con group. This indicates that alcohol metabolism triggered a substantial release of proinflammatory factors in the livers of ALD mice, activating the inflammatory pathway and resulting in liver inflammation. CA intervention restored the inflammatory factors to their normal levels, suggesting the effective inhibition of alcohol-induced liver inflammation by CA. Sil can also restore the levels of inflammatory factors to a certain extent, but its recovery level is not as good as CA. Therefore, we will focus on studying the anti-inflammatory effects of CA.

To further study the inhibitory effects of CA on inflammation in ALD mice, relevant investigations were performed at the protein level. We examined csapase 1, Cleaved-caspase 1, caspase 3 and Cleaved-caspase 3 (Figure 5B). There was an increase in the protein expression of Cleaved-caspase 1, 3, and a decrease in the protein expression of caspase 1, 3, in the EtOH group compared to the Con group. The decreased protein expression of Cleaved-caspase 1, 3, in the EtOH+CA group compared to the EtOH group suggests that CA has an inhibitory effect on inflammation-induced cellular pyroptosis. The NF-κB signaling pathway is a widely known inflammation-related pathway. We detected the key proteins in this pathway (MYD88, TLR4, p65, and p-p65) using WB analysis (Figure 5C). The NF-κB p-p65, MYD88, and TLR4 expression levels in mouse liver tissue were significantly higher in the EtOH group than those in the Con group, indicating the activation of the NF-κB pathway in ALD mouse livers and the release of numerous inflammatory factors. CA can effectively inhibit the expression of NF-κB p-p65, MYD88, and TLR4. IF analysis of NF-κB was performed to gain further insights into the effect of CA on the hepatic NF-κB pathway in ALD mice. Figure 5D shows that the red fluorescence intensity, which corresponded to NF-κB, was significantly higher in the liver tissue of mice in the EtOH group compared with those of the Con group. This indicated the activation of the NF-κB pathway ALD mouse livers, resulting in liver inflammation. In contrast, CA effectively attenuated NF-κB fluorescence intensity, indicating the effective inhibition of hepatic inflammation. This was consistent with the WB results. CA inhibited the NF-κB pathway and attenuated NF-κB, p-p65, MYD88, and TLR4 expression, suggesting that CA could effectively inhibit NF-κB p65 phosphorylation, thereby suppressing NF-κB pathway activation, reducing the inflammatory cascade, and inhibiting hepatic inflammation.

### 2.6. Molecular Docking Suggested That CA Interacts with Several Key Proteins in the NF-κB/AMPK Signaling Pathway

**Figure 6:**
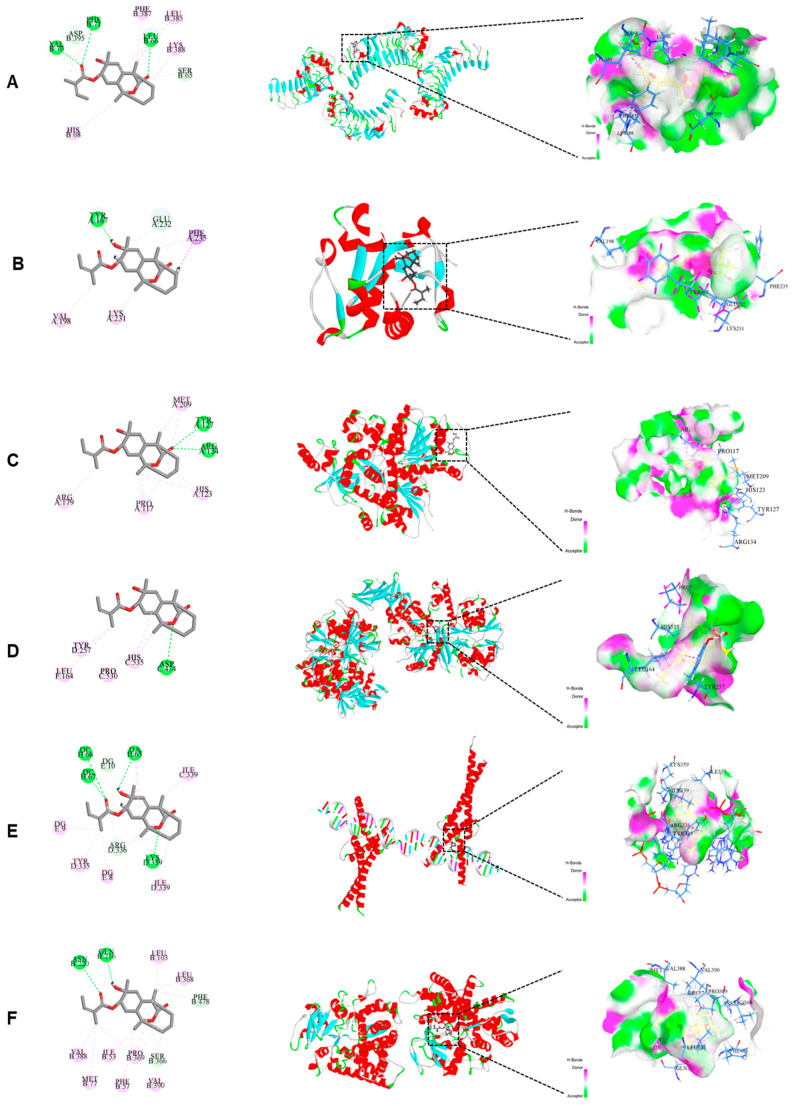
Binding of CA to various target proteins and the corresponding three-dimensional hydrogen-binding mode interactions. (**A**) TLR4, (**B)** MYD88, (**C**) NF-κB, (**D**) AMPK, (**E**) SREBP-1c, and (**F**) CYP2E1.

Molecular docking was used to predict and evaluate the interactions between CA and six key protein targets within the inflammatory (TLR4, MYD88, and NF-κB) and lipid metabolic (AMPK, SREBP-1c, and cytochrome P450 2E1 (CYP2E1)) pathways. The docking results are shown in Table 1. The ligand molecules were mainly bound to the receptor proteins through hydrogen, π-alkyl, and carbon-hydrogen bonds. All LibDock scores were >100, indicating strong binding between CA and the target proteins TLR4, MYD88, NF-κB, AMPK, SREBP-1c, and CYP2E1. The molecular docking models of CA and the target proteins are shown in Figure 6. Among these, SREBP-1c achieved a docking score of 116.711. Its docking model involved four amino acid residues (DCH66, DCH67, DAH65, and LYSD359) that formed hydrogen bonds. MYD88, NF-κB, AMPK, SREBP-1c, and CYP2E1 are key signaling pathway proteins associated with inflammation and lipid metabolism. We hypothesized that CA could be a potential agent for the prevention and treatment of ALD.

### 2.7. Effect of CA on Alcohol-Induced AML-12 Cell Survival and Associated Levels of Inflammation

**Figure 7:**
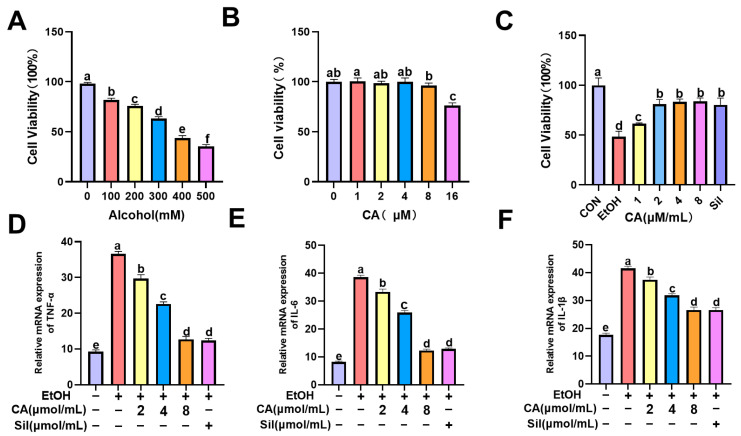
Detection of AML-12 cell viability by CCK-8 method. (**A**) Effect of different concentrations of alcohol on cell viability. (**B**) Effect of different concentrations of CA on cell viability. (**C**) Effect of CA on alcohol-induced cell protection. (**D**) mRNA expression of TNF-α (**E**) IL-6 (**F**) IL-1β. Data are expressed as mean ± standard deviation. Different letters (a–f) represent significant differences among groups (*p* < 0.05, *n* = 3), determined by one-way ANOVA using Duncan′s post-test.

In previous animal experiments, the intervention of CA alleviated the symptoms of inflammation in the liver. Mouse liver transcription data also showed that CA may play a protective role in ALD by affecting Toll-like receptor and NF-κB-related pathways. TLR4 is an important member of the Toll-like receptor family and an upstream key target of the NF-κB signaling pathway. In order to further explore the protective mechanism of CA on ALD in mouse normal hepatocytes, AML-12 was studied by alcohol-induced hepatocyte injury, and the protective mechanism of CA on hepatocytes in an in vitro ALD model was explored. The 24-h viability of AML-12 cells stimulated with ethanol at different concentrations (100–500 mM) was determined by CCK-8 assay. As shown in Figure 7A, AML-12 cells exhibit moderate toxicity and >50% cell viability at an alcohol concentration of 300 mM; therefore, an alcohol concentration of 300 mM was chosen for subsequent dosing. As shown in Figure 7B, 0–8 μg/mL of CA had no obvious toxic effect on cells. In addition, CA was tested to have a protective effect on alcohol-induced AML-12 cell damage. As shown in Figure 7C, the results showed that the CA concentration in the range of 2–8 μM/mL significantly improved the viability of AML-12 cells. Based on the above results, 300 mM ethanol was selected as the optimal dose for inducing cell damage, and 2, 4 and 8 μM/mL CA were used as the low, medium and high doses and 10 μg/mL Sil as the optimal doses for further experimental studies. The mRNA transcription levels of inflammatory cytokines TNF-α, IL-6 and IL-1β in alcohol-induced AML-12 cells were detected by qRT-PCR. The results showed that alcohol could significantly cause the transcription of inflammatory cytokines TNF-α, IL-6 and IL-1β in AML-12 cells, and the transcriptional activities of TNF-α, IL-6 and IL-1β were significantly reduced and dose-dependent after CA intervention, as shown in Figure 7D–F.

### 2.8. Effect of CA on Alcohol-Induced Inflammatory Pathway-Related Proteins in AML-12 Cells

**Figure 8:**
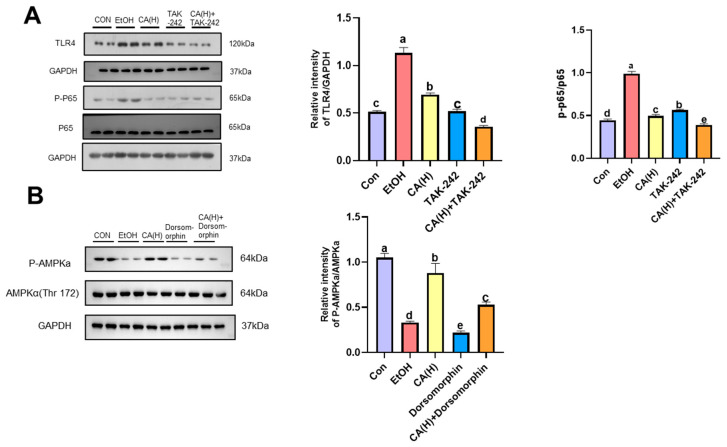
Effect of CA on inflammatory pathway proteins and AMPK in alcohol-induced AML-12 cells. (**A**) TLR4/NF-κB Western Blot Representative Image. (**B**) AMPK Western Blot Representative Image. Data are Con expressed as mean ± standard deviation. Data are expressed as mean ± standard deviation. Different letters (a–e) represent significant differences among groups (*p* < 0.05, *n* = 3), determined by one-way ANOVA using Duncan′s post-test.

To evaluate TLR4/NF-κB pathway activation, we employed pharmacological inhibition using TAK-242, a well-characterized TLR4 inhibitor known to specifically suppress TLR4. Western blot analysis (Figure 8A) showed that compared with the Con group, the protein expression of TLR4 and p-p65/p65 in AML-12 cells increased after alcohol induction, decreased after high-dose CA intervention, and significantly decreased after the addition of TLR4 inhibitors, which was consistent with the previous in vivo results. These results indicated that CA could alleviate alcohol-induced hepatocyte inflammation by inhibiting the expression of TLR4/NF-κB pathway protein.

### 2.9. CA Alleviates Alcoholic Liver Injury by Activating AMPK

To evaluate the effect of CA on AMPK activation, we employed pharmacological inhibition using dorsomorphin, a well-characterized AMPK inhibitor known to specifically suppress AMPK activity. WB analysis (Figure 8B) revealed that, compared to the control group, p-AMPK protein expression decreased in AML-12 cells following alcohol induction. This decreased expression increased after high-dose CA intervention but was significantly reduced upon addition of the AMPK inhibitor. However, AMPK expression levels increased following CA plus AMPK inhibitor treatment. This indicates that CA can activate AMPK. These results suggest that CA may alleviate lipid accumulation in alcoholic liver injury by inhibiting AMPK activation.

### 2.10. CA Regulated Gut Microbiota

**Figure 9:**
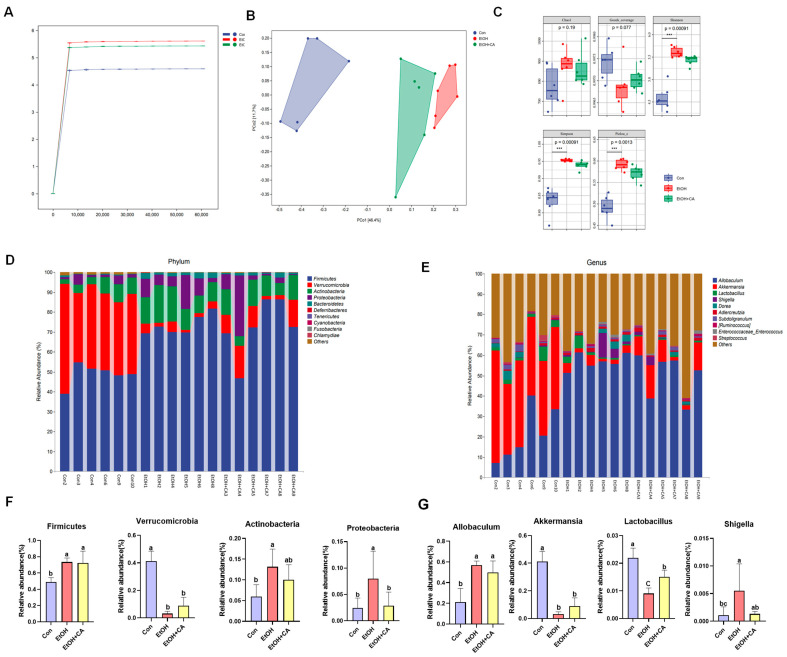
Impact of CA intervention on fecal microbiota structure and function. (**A**) Rarefaction curve and Shannon diversity index. (**B**) Principal coordinates analysis based on the Bray–Curtis distances algorithm. (**C**) Alpha diversity analysis of intestinal microbiota in mice. (**D**) Relative abundance of gut microbiota at the phylum level, with different letters indicating significant differences among different groups. (**E**) Relative abundance of gut microbiota at the genus level, with different letters indicating significant differences among groups. (**F**) Phylum and (**G**) levels represent changes in colony abundance. Data are expressed as mean ± standard deviation. Different letters (a–c) represent significant differences among groups (*p* < 0.05, *n* = 3), determined by one-way ANOVA using Duncan′s post-test.

In order to explore the effect of gut microbiota on liver disease, 16S rRNA gene sequencing was performed on the feces of ALD mice, and the data were analyzed by the Payson Wakefield gene cloud platform to explore the effect of CA on intestinal microbiota. Rank abundance curves were used to explain species richness and community evenness. As shown in Figure 9A, the Shannon index curves for all three groups flattened at extracted sequences >10,000. The OTU tended to saturate, indicating a reasonable amount of experimental data measurements and a sufficient amount of sequencing data to reflect the vast majority of microbial diversity information in the samples.

Beta diversity, also known as between-habitat diversity, refers to the dissimilarity of species composition or the rate of species turnover between communities that vary along an environmental gradient. The distance between two sample points represents the dissimilarity between those samples. The closer the two sample points are, the more similar the species compositions of the corresponding samples. The gap between the EtOH and Con groups was relatively large, while the sample points of the EtOH+CA and Con groups were closer, indicating the similarity between the intestinal flora of the EtOH+CA and Con groups (Figure 9B). Alpha diversity, also known as within-habitat diversity, is indicative of species richness, diversity, and evenness in a locally homogeneous habitat. The alpha diversity of the microbial community was assessed more comprehensively using the following indices: the Chao1 index, characterizing richness; the Shannon and Simpson indices, characterizing diversity; Pielou’s evenness index, characterizing evenness; and Good’s coverage index, characterizing cover. Alpha diversity analysis of microorganisms in fecal samples between groups is presented in Figure 9C.

We then analyzed the gut microbiota at the phylum and genus levels. Compared with the Con group, the EtOH group exhibited an increase in Firmicutes, Actinobacteria, and Proteobacteria, and a decrease in Verrucomicrobia (Figure 9D). Four dominant bacterial genera were selected for group comparisons. (Figure 9E). Compared with the Con group, the EtOH group showed a significant increase in *Allobaculum* and a significant decrease in *Akkermansia*. Compared with the EtOH group, the EtOH+CA group exhibited an increased abundance of *Akkermansia* (Figure 9F,G).

**Figure 10:**
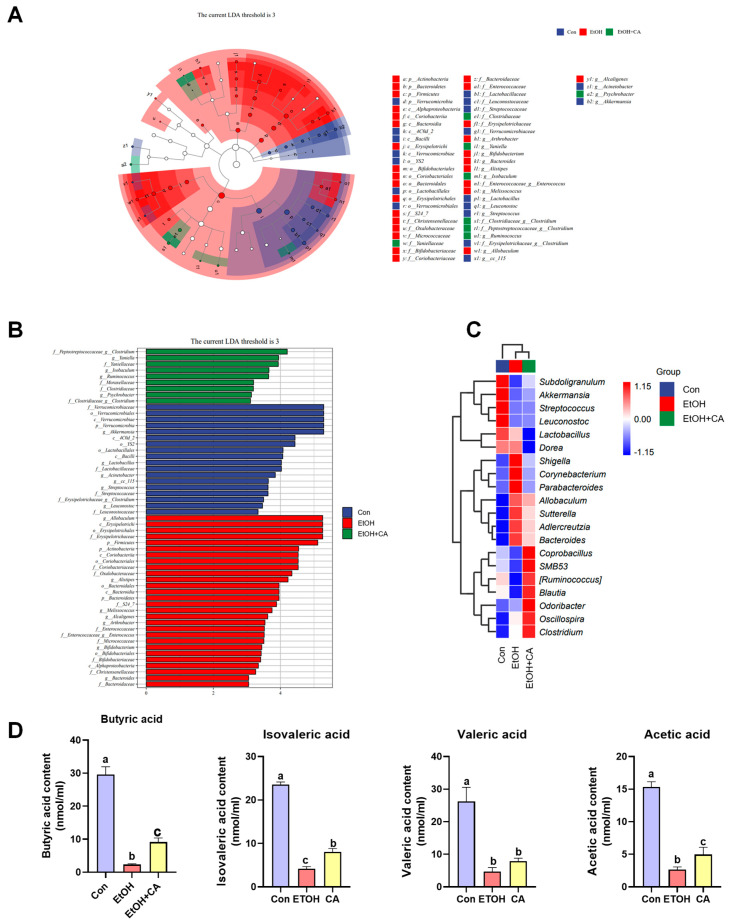
SCFA levels in mouse feces. (**A**) LEfSe cladogram from the phylum to genus levels. (**B**) LDA bar plot based on the Kruskal–Wallis sum-rank test. (**C**) Community heatmap analysis and (**D**) SCFA levels in mouse feces. Data are expressed as mean ± standard deviation. Different letters indicate significant differences among groups (*n* = 3). Different letters (a–c) represent significant differences among groups (*p* < 0.05, *n* = 3), determined by one-way ANOVA using Duncan’s post-test.

We performed linear discriminant analysis effect size (LEfSe) on the Con, EtOH, and EtOH+CA groups and obtained linear discriminant analysis (LDA) scores and a classification diagram (Figure 10A,B). *Akkermansia* had the highest colony abundance, *Allobaculum*, and *Peptostreptococcaceae clostridium*, corresponded to the Con, EtOH, and EtOH+CA taxa, respectively. An analysis of the taxonomic composition of the genus (Figure 10C) showed that the beneficial bacterium *Akkermansia* was significantly downregulated in the EtOH group compared with the Con group and upregulated in the EtOH+CA group compared with the EtOH group. We observed the addition of some acid-producing genera to the EtOH+CA group, including *SMB53*, *Ruminococcus*, *Odoribacter*, and *Oscillospira*.

### 2.11. Effect of CA on the Production of Short-Chain Fatty Acids

The intestinal SCFA levels of the mice were studied next (Figure 10D). The mice in the EtOH group exhibited reduced levels of butyric acid, isovaleric acid, valeric acid, and acetic acid compared with the Con group. However, CA supplementation increased SCFA production. Thus, SCFA production and the growth of the associated gut flora were inhibited in the EtOH group, which was reversed by CA.

## 3. Discussion

In this study, Through transcriptomics and molecular docking, it was discovered that CA can alleviate chronic alcohol-induced hepatic steatosis, reduce inflammation, and modulate the gut microbiota. Transcriptomic findings suggested CA may act via modulating the AMPK/NF-κB pathway. Consequently, we first validated this mechanism in vivo, demonstrating CA’s capacity to regulate AMPK, NF-κB, and their associated tight junction proteins. Subsequently, we verified CA’s effects on AMPK and inflammatory signaling pathways in vitro using the TLR4 inhibitor (TAK-242) and AMPK inhibitor (Dorsomorphin) respectively.

As a highly prevalent chronic liver disease worldwide, alcoholic liver disease (ALD) represents a leading cause of mortality associated with hepatic pathologies [17]. In its early stages, ALD manifests as relatively mild and reversible conditions such as fatty liver and alcoholic hepatitis. However, without timely intervention, the disease can progress to more severe forms, including fibrosis and cirrhosis [18]. Research shows [19], key mechanisms implicated in ALD pathogenesis include inflammation, oxidative stress, lipid accumulation, and gut microbiota dysbiosis. In this study, alcohol-exposed mice exhibited significant increases in serum ALT, AST, MDA, TG, and TC levels, as well as elevated liver index (*p* < 0.05), indicating hepatocellular damage and disrupted lipid metabolism. These alterations were effectively reversed by CA administration. Histopathological examination via H&E and Oil Red O staining revealed substantial lipid vacuolation, lipid droplet accumulation, and inflammatory infiltration in the ethanol group, all of which were markedly attenuated by CA treatment. This is supported by observations that treatment with CA led to improved liver function, alleviated hepatic acidosis, suppressed inflammatory responses, and restored gut microbial balance in patients.

Transcriptomic analysis revealed that CA alleviates alcohol-induced liver injury by modulating lipid accumulation and mitigating inflammation. Further Western blot experiments confirmed that its core mechanism lies in activating the AMPK signaling pathway and suppressing TLR4 expression. Research shows that EtOH has a significant effect on the expression of proteins related to lipid metabolism and the accumulation of TG (triglycerides) in the liver, a process that in turn triggers alcoholic fatty liver injury [20]. Specifically, ethanol intake interferes with normal lipid metabolic pathways in the liver, leading to excessive accumulation of fat in hepatocytes. AMPK (adenylate-activated protein kinase), a key metabolism-regulating enzyme, is extensively involved in lipid metabolic processes [21]. It negatively regulates lipid production by phosphorylating and inhibiting key lipogenic enzymes from head lipid synthesis. In addition, the AMPK/SREBP signaling pathway plays an important role, which further regulates the expression of lipid synthesis-related genes by affecting the activity of cholesterol regulatory element binding proteins (SREBPs) [22]. In the context of alcoholic fatty liver, AMPK activity may be inhibited, leading to its inability to effectively inhibit lipid synthesis, thereby exacerbating fatty liver formation and progression. Therefore, an in-depth understanding of the role of the AMPK/SREBP signaling pathway in ethanol-induced lipid metabolism disorders [23] is needed. The AMPK signaling pathway is also vital for improving lipid metabolism disorders. Our research shows that CA significantly ameliorated EtOH-induced hepatic steatosis. Importantly, CA caused AMPK phosphorylation, indicating its ability to counteract EtOH-induced hepatic steatosis via AMPK activation. In the liver, SIRT1 is essential for the balance of glucose, lipids, and cholesterol [24]. Fatty liver is caused by abnormalities in hepatic lipid metabolism, and activation of SIRT1 plays an important role in this process by inhibiting de novo lipogenesis and increasing β-oxidation of fatty acids [25]. SIRT7 reduces lipid accumulation by inhibiting the expression of key genes for fatty acid synthesis. CA can upregulate SIRT1 and SIRT7 to regulate lipid accumulation. CA also significantly inhibits CYP2E1 and upregulates antioxidant genes and hepatic glutathione. SREBP homologue SREBP-1c is a nuclear transcription factor involved in lipid metabolism and plays an important role in fatty acid and TG synthesis. Alcohol exposure increases lipogenesis through the upregulation of SREBP-1c and its target lipogenesis-related genes, leading to hepatic steatosis [26]. WB and IHC analysis showed that CA inhibited the expression of SIRT1 and SREBP-1c, resulting in reduced lipid accumulation.

ALD is a metabolic liver disease whose pathological progression is largely determined by the inflammatory response [27]. Upon alcohol exposure, the expression levels of Cleaved-caspase 1 and 3 are elevated but reduced by CA, the pathogenesis of ALD is dependent on liver inflammation, and LPS activates TLR4, which upregulates downstream genes such as NF-κB and MYD88 [28]. Activation of these pro-inflammatory genes produces deleterious inflammatory cytokines that trigger inflammation and liver damage. Previous studies have shown that SIRT1 plays an important role in regulating lipid metabolism and suppressing inflammation by inhibiting NF-κB-mediated transcription [29]. Although SIRT1 and the NF-κB pathway have co-evolved to maintain homeostasis in vivo, an antagonistic relationship exists between the two. Importantly, SIRT1 interacts directly with NF-κB by deacetylating the p65 subunit of NF-κB, causing translocation of NF-κB from the nucleus to the cytoplasm and inhibiting its transcriptional activity. In the EtOH group, the expression of SIRT1 was significantly decreased and the expression of NF-κB was significantly increased, which was alleviated in the CA group. CA significantly down-regulated TLR4 and its downstream inflammatory mediators, resulting in a significant decrease in the levels of TNF-α, IL-1β, IL-6 and IL-18 in the mouse liver compared to the ethanol group.

Dysregulation of the gut microbiome is considered a key aspect of ALD due to the intimate physiological relationship between the gut and the liver. Maintaining a state of intestinal symbiosis, stabilizing the intestinal mucosal barrier and preventing cellular responses to microbial products are protective factors against ALD; therefore, the gut microbiota appears to play an important role in the pathogenesis of ALD. Numerous studies have confirmed a close association between liver disease and the gut microbiota [30,31]. *Acemansia muciniphila* is a promising candidate probiotic as an intestinal commensal living in the mucosal layer. *A. muciniphila* plays an important role in improving metabolic function and immune responses in the host, and its role in fatty acid metabolism contributes to the maintenance of intestinal barrier function [32]. We found that CA increased the relative abundance of *Acemansia* in the gut and therefore increased the expression of *Acemansia*, which ameliorated alcohol-induced intestinal barrier dysfunction and consequently prevented ALD. *Shigella spp.* are Gram-negative bacilli, one of the most important intestinal pathogens affecting humans [33]. Shigella was also positively correlated with hepatic inflammatory factors. In addition, CA intervention significantly increased the numbers of SMB53 and *Ruminococcus*, suggesting that CA modulates the number of beneficial bacteria, thereby alleviating ALD. Previous studies have also shown that certain genera of bacteria, including SMB53, Ruminococcus, Odoribacter, and Oscillospira, produce SCFAs [34,35]. The presence of SMB53, *Ruminococcus*, *Odoribacter*, and *Oscillospira* has also been shown to reduce the production of SCFAs [36]. Decreases in *Odoribacter* populations have been associated with several microbiologically related diseases. Vibrio spp. frequently appear in high-throughput sequencing results and are abundant in the animal and human gut [37]. Therefore, CA may prevent dysbiosis in the gut microbiota of ALD patients.

SCFAs constantly influence host immunity and metabolism [38]. To protect hepatocytes from harmful metabolites and pathogen-associated patterns, it is crucial to maintain the integrity of the intestinal mucosa [39]. The liver may be exposed to a broad spectrum of harmful metabolites that promote liver inflammation due to gut flora dysbiosis and increased intestinal permeability. *Clostridium butyricum* is a potential probiotic that produces butyric acid and has been shown to limit liver steatosis, restore intestinal barrier function, and improve liver inflammation [40]. SCAF production and the growth of the associated gut flora were inhibited in the EtOH group; CA intervention restored SCAF production. The abundance of flora capable of producing SCFAs increased in response to CA. This recovery was particularly evident at the butyric acid level and consistent with the earlier results regarding the gut flora.

Using alcohol-induced AML-12 cell damage as a model, the results showed that CA had little toxicity to AML-12 cells, and reduced the mRNA expression of TNF-α, IL-6 and IL-1β inflammatory factors in alcohol-induced AML-12 cells to varying degrees in dose-dependence. The inflammatory response to ALD may further exacerbate alcohol-induced hepatocyte death, intestinal bacterial dysbiosis, and oxidative stress [41]. Pro-inflammatory factors have a variety of pathogenic effects in ALD, including their ability to induce the production of pro-inflammatory cytokines, sensitize hepatocytes to apoptosis signals, disrupt fatty acid synthesis pathways, induce hepatic steatosis, and promote liver fibrosis [42]. TLR4 pathway is considered to be one of the important signaling pathways involved in inflammation, and TAK-242 is used as a TLR4 inhibitor [43], and after the addition of TAK-242 in the WB test in vitro, the protein levels of TLR4 and p-NFκB/p-NFκB were significantly reduced compared with the Con group, and it was found that CA could reduce the occurrence of inflammatory response by regulating the TLR4/NF-κB pathway, thereby playing a protective role in the liver of ALD mice. As an important pathway regulating lipid accumulation, AMPK was investigated to verify the activation effect of CA. In vitro, dorsomorphin was employed as an AMPK inhibitor [44]. Western blot analysis confirmed that CA could still activate AMPK in the presence of the inhibitor, indicating that CA activates AMPK to alleviate lipid accumulation and thereby mitigate alcoholic liver disease.

In conclusion, the protective effect of CA against hepatic steatosis and inflammatory lesions induced by chronic alcohol could be linked to its modulation of intestinal flora and the AMPK/NF-κB pathway. In subsequent experiments, the interaction between CA and gut microbiota could be systematically investigated using techniques such as fecal microbiota transplantation; meanwhile, other liver injury models, those induced by CCl_4_ or TAA in mice could be employed to further validate the hepatoprotective effects of CA. These studies will help establish a solid theoretical foundation for the development of CA as a novel plant-derived hepatoprotective agent.

## 4. Materials and Methods

### 4.1. Materials

CA was obtained through laboratory isolation and recrystallization from methanol, resulting in white CA crystals with a purity of up to 98%. The CA structure (Figure 1A) was determined using nuclear magnetic resonance and high resolution electrospray ionization mass spectroscopy. ^1^H NMR and ^13^C NMR spectra of CA are shown in Figures S1 and S2. Lieber-DeCarli control (TP4030C) and alcoholic (TP4030D) liquid diets were purchased from Nantong Trophy Co., Ltd. (Nanjing, China). Silibinin (Sil) was purchased from Tasly Shengte Pharmaceutical Co., Ltd. (Tianjin, China). Antibodies were purchased from Beyotime Biotechnology (Shanghai, China). Acetic acid, propionic acid, n-valeric acid, and isovaleric acid were purchased from Aladdin Industries (Shanghai, China).

### 4.2. Animal Experiments

Female C57BL/6 mice (8 weeks old, 20–22 g) were purchased from Changsheng Biotechnology Co., Ltd. (Liaoyang, China; Quality Certificate No.: 210726221101935241). The animal experiments were approved by the Institutional Animal Care and Use Committee of Shenyang Pharmaceutical University (SPYU-IACUC-S2022-11.30-104) in accordance with the guidelines of the “Regulations for the Administration of Laboratory Animals” issued by the National Science and Technology Commission. All mice were housed in a sterile room with a 12-h light/dark cycle.

The mice were acclimatized for 7 d, then randomly assigned to four groups (*n* = 10): control (Con); ethanol (EtOH); EtOH+CA(L) (20mg/kg body weight (BW)/d); EtOH+CA(H) (40mg/kg body weight (BW)/d); and EtOH+Sil (100 mg/kg BW/d). Our dose selection was selected based on our previous experiments [45]. The mice in the EtOH, EtOH+CA(L), EtOH+CA(H, and EtOH+Sil groups were fed a Lieber-DeCarli ethanol liquid diet containing 5% EtOH (EtOH-fed), while those in the Con group were fed a control diet (CD-fed). The mice in the EtOH+CA group were administered CA via gavage. Sil was administered by gavage to mice in the EtOH group as a positive control. After 11 d, the EtOH-fed mice were administered a single dose of 31.5% alcohol (5 g/kg) by gavage, while those in the Con group were administered 5g/kg of 45% maltodextrin. The mice were fasted for 9 h, anesthetized, had their eyeballs removed for blood collection, and were euthanized by cervical dislocation. The total sera, gut contents, and liver tissue samples were stored at −80 °C for subsequent analysis. The liver index was calculated as follows:

liver index = liver wet weight/BW × 100.

### 4.3. Serum and Liver Biochemical Markers

Serum samples are obtained by centrifuging whole blood at 3000 rpm for 10 min at 4 °C. The following liver function parameters were measured using the appropriate test kits according to the manufacturer’s instructions: alanine aminotransferase (ALT; C009-2-1), aspartate aminotransferase (AST; C010-2-1), total cholesterol (TC; A111-1-1), triglycerides (TG; A110-1-1), superoxide dismutase (SOD; A001-1), malondialdehyde (MDA; A003-1), and glutathione peroxidase (GTP).), malondialdehyde (MDA; A003-1) and glutathione peroxidase (GSH-Px; A005) (Nanjing, China). In addition, tumor necrosis factor α (TNF-α), interleukin 1β (IL-1β), interleukin 6 (IL-6) and interleukin 18 (IL-18) levels were measured using enzyme-linked immunosorbent assay (ELISA) kits purchased from Servicebio (Wuhan, China).

### 4.4. Histopathological

Livers were fixed in 4% paraformaldehyde, dehydrated in ethanol, embedded in paraffin, and sectioned at a thickness of 4 μm. Sections were stained with hematoxylin and eosin (H&E) and imaged using a light microscope (Nikon Eclipse E100, Tokyo, Japan). Fresh liver tissue samples were frozen, sectioned and immersed in Oil Red O solution. After restaining, the sections were observed by light microscopy (Nikon, Tokyo, Japan).

### 4.5. Transcriptomic

Total RNA content was extracted from liver tissue samples using TRIzol^®^ reagent (Invitrogen, Carlsbad, CA, USA). Extracted RNA concentration and mass were determined spectrophotometrically and by 2100 Bioanalyzer System (Agilent Technologies, Santa Clara, CA, USA), respectively. Libraries were prepared using the TruSeq RNA Sample Preparation Kit (Illumina, San Diego, CA, USA). RNA sequencing was performed using the Novaseq 6000 system. Contamination-free clean reads were obtained using FASTP (https://github.com/opengene/fastp) (accessed 25 October 2024) and then localized using HISAT2 (https://daehwankimlab.github.io/hisat2/, accessed 29 October 2024). Gene expression levels were expressed as per kilobase per million fragments. Analyses were performed on a free online platform provided by Personalbio Cloud Platform (Personalbio.cn). Expression analyses were performed on high-quality clean data using DESeq2 (version 1.20.0).

### 4.6. Enzyme-Linked Immunosorbent

Liver tissues were thawed, homogenized with cold PBS (1:9, *w/v*), centrifuged at 3000 rpm for 20 min, and ELISA was performed to detect TNF-α, IL-1β, IL-6, and IL-18.

### 4.7. Immunohistochemistry (IHC)

Paraffin sections are deparaffinized, blocked with 3% Bovine Serum Albumin (BSA) for 30 min and incubated overnight at 4 °C with Phosphorylated AMPKα Rabbit Polyclonal Antibody (1:200) and SIRT1 Rabbit Polyclonal Antibody (1:200). The appropriate secondary antibody is then added and incubated at room temperature for 50 min away from light. Diaminobenzidine staining is used to produce a brown stain. Sections are stained with hematoxylin for approximately 3 min, rinsed with tap water, placed in hematoxylin differentiation solution for a few seconds, rinsed with tap water, returned to hematoxylin blue solution, and then rinsed with running water. After dehydration and sealing, the sections were illuminated with white light and observed under a light microscope (Nikon, Tokyo, Japan, E100).

### 4.8. Molecular Docking

Molecular docking was performed using the Discovery Studio 2016/LibDock protocol to explore the binding patterns of CA to proteins in the NF-κB and AMPK signaling pathways. The fractions calculated by LibDock are used to examine the interactions between CAs and related pathway proteins. A score of >100 indicates potential targeted activity.

### 4.9. Cell Culture

AML12 cells were cultured in a cell culture incubator at 37 °C with 5% CO_2_ using DMEM high glucose medium. Cell recovery: Remove frozen cells from the liquid nitrogen tank and thaw. Centrifuge the cells at 1100 rpm for 5 min, discard the supernatant and incubate in fresh medium. Cell Passaging: Cells are passaged when the cell confluence is 80–90%. Remove the medium from the original dish, transfer it to a new dish, pre-mix it and incubate it in the incubator. Cell freezing and preservation: Freeze and preserve the excess cells in good condition during the cell culture process.

### 4.10. Determination of Cell Viability

When the cells grew to logarithmic growth, AML12 cell suspension was inoculated in 96-well plates for 24 h, and then treated with different concentrations of alcohol (100, 200, 300, 400, and 500 mM) for 24 h. Cell viability was determined by the CCK-8 method. Six replicate wells per group were incubated with 10 μL of CCK8 solution for 4 h. Excess culture was removed from the wells. Excess medium was removed from the wells, 100 μL of DMSO was added, and the absorbance at 450 nm of each well was measured using an enzyme marker to determine the alcohol modeling dose. Meanwhile, the optimal concentrations of CA (1, 2, 4, and 8 μm) were studied for 24 h, and then incubation was continued for 24 h after exposure to the previously determined alcohol modeling conditions. Cell viability (OD administered group/OD blank group (100%).

### 4.11. Immunofluorescence (IF)

Paraffin sections were deparaffinized, blocked with 3% BSA for 30 min, and incubated with NF-κB p65 (1:200) overnight at 4 °C. Suitable secondary antibodies were added for 50 min at room temperature, and DAPI Dye solution was added for 10 min at room temperature. Then the appropriate secondary antibody was added and incubated in the dark at room temperature for 50 min. DAPI Dye solution was added and incubated in the dark at room temperature for 10 min. Slides were immersed in phosphate buffered saline (PBS) (pH 7.4) and washed three times for 5 min each on a decolorizing shaker. Autofluorescent bursting agent B solution was added for 5 min and rinsed under running water for 10 min. The sections were then imaged with a fluorescence microscope (Nikon, Tokyo, Japan, Nikon ECLIPSE C1).

### 4.12. Western Blot (WB)

Liver tissue was lysed in a grinder using radioimmunoprecipitation and phenylmethylsulfonyl fluoride buffer. The homogenate was kept on ice for 30 min and centrifuged at 12,000 rpm for 10 min at 4 °C to collect the supernatant. Standard protein concentrations were determined using a bicinchoninic acid assay kit purchased from Servicebio (Wuhan, China) and Sodium Dodecyl Sulfate (SDS) Protein Sampling Buffer (Servicebio (Wuhan, China)) was added to quantify the protein concentration of all samples. Protein samples were separated by SDS polyacrylamide gel electrophoresis and transferred to nitrocellulose filter membranes, which were then blocked with 5% skim milk for 1 h. The strips were incubated with horseradish peroxidase-labeled goat anti-rabbit/mouse antibody (1:1000) for 1 h, then rinsed with TBST, and Erythrina cristagilli lectin fluorescein was added to visualize protein expression on the membrane.

### 4.13. 16S rRNA Amplicon Sequencing of Gut Microbiota

For 16S rRNA sequencing, cecal and colonic contents from each group (*n* = 10) were pooled. Commercial kits (Omega Bio-tek, Norcross, GA, USA) were used to extract DNA from pooled samples. Electrophoresis and spectrophotometry (NanoDrop 2000, Thermo Fisher Scientific, Wilmington, NC, USA) were then used to assess the integrity and purity of the extracted DNA. Polymerase chain reaction experiments were performed by Paysonol Co. Ltd. (Shanghai, China). Ltd. (Shanghai, China). Primers 338F (ACTCCTACGGAGGCAGCAGCA) and 806R (GGACTACHACHVGGGGTWTCTAAT) were used to amplify regions V3–V4 of the 16S rRNA gene. PCR products were sequenced using a Novaseq platform (Illumina). Sequence analysis was performed with QIIME2 and was based on the overlap ratios of the original sequencing data. Analyses performed included operational taxonomic unit (OTU) clustering and species annotation, alpha diversity analysis, beta diversity analysis, phylogenetic beta diversity analysis, phylogenetic testing, and taxonomic species composition analysis.

### 4.14. Measurement of Intestinal Short-Chain Fatty Acids (SCFAs)

The procedure began by mixing 100 mg of feces with 1 mL of ultrapure water. It was homogenized at 70 Hz for 180 s and centrifuged at 12,000 rpm for 10 min at 4 °C. The supernatant was pipetted into a 1.5 mL tube and homogenized by adding 20 μL of the internal standard solution and 50 μL of 50% hydrochloric acid for 20 s. The pH of the feces solution was then adjusted to 1.0. The acidified feces homogenate was extracted with ether (570 μL), vortexed for 3 min at 4 °C and centrifuged for 10 min at 12,000 rpm. The extracted sample containing ether layer (300 μL) was subjected to gas chromatography–mass spectrometry (GC–MS). An 8890 GC system connected to a 5977B mass spectrometer (Agilent Technologies, Santa Clara, CA, USA) was used for GC-MS analysis. The chromatographic column was an Agilent HP-1701 with an injection temperature of 230 °C, an ion source temperature of 230 °C, a quadrupole column temperature of 150 °C, and an interface temperature of 250 °C. The chromatographic separation was carried out on an 8890 GC system connected to a 5977B mass spectrometer (Agilent Technologies, Santa Clara, CA, USA). The carrier gas flow rate was kept at 1.5 mL/min, and 1 μL of the extracted sample was automatically injected in split mode (10:1 split ratio). The following temperature program was used: 50 °C hold for 5 min, 5 °C/min up to 150 °C, 20 °C/min up to 160 °C, 160 °C hold for 2 min, 20 °C/min up to 260 °C, 260 °C hold for 2 min. The instrument was operated in electroshock mode with an ionization energy of 30 eV and a scanning range of m/z 30–600. The SCFA content was quantified using an external standard and expressed in nmol/mL feces.

### 4.15. Statistical Analysis

Statistical analysis was performed using GraphPad Prism (version 9; San Diego, CA, USA). Statistical analysis was conducted based on one-way ANOVA. Values are expressed as mean ± standard deviation (SD). *p* < 0.05 was considered statistically significant.

## 5. Conclusions

In summary, CA can prevent alcoholic liver disease by inhibiting the TLR4/MYD88/NF-κB pathway and regulating the gut microbiota. Providing new insights into the multipurpose of Callistephus chinensis, these findings suggest that CA has the potential to further develop as a promising alternative for the treatment of ALD.

## Figures and Tables

**Table 1:** CA-target protein interaction.

Number	Protein	PDB ID	LibDock Score	H-Bonds Number	H-Bonds Residues
1	TLR4	2Z66	106.344	3	VALB70, PHEB71, LEUB66
2	MYD88	4DOM	100.359	1	TYRA167
3	NF-κB	6SH6	105.696	2	TYRA127, ARGA134
4	AMPK	7YMJ	109.448	1	ASPC474
5	SREBP-1c	1AM9	116.711	4	DCH66, DCH67, DAH65, LYSD359
6	CYP2E1	3E6I	104.621	2	ASNB220, GLNB216

## Data Availability

All data are contained within the article.

## Supplementary Materials

The following supporting information can be downloaded at: https://www.mdpi.com/article/10.3390/molecules30224371/s1, Figure S1: ^1^H NMR of CA, Figure S2: ^13^C NMR of CA.
