# Supplementary material for: A Novel Sesquiterpene from Callistephus chinensis Improves Alcohol-Induced Liver Disease by Regulating the AMPK/NF-κB Signaling Pathway and Gut Flora
Source: Molecules. 2025 Nov 12;30(22):4371. doi: 10.3390/molecules30224371 (PMC12655352; doi:10.3390/molecules30224371)
Supplement: Supplementary file 1 [file molecules-30-04371-s001.zip › Supplementary Material.pdf]

Supplementary Material

A Novel Sesquiterpene from *Callistephus chinensis* Improves Alcohol-Induced Liver Disease by Regulating the AMPK/NF-κB Signaling Pathway and Gut Flora

Bingxin Zhang †, Ning Wang †, Xiaoxu Chen, Nan Yang, Ying Zhao * and Xiaoshu Zhang *

School of Functional Food and Wine, Shenyang Pharmaceutical University, Shenyang 110016, China; 13177089059@163.com (B.Z.); 15094062128@163.com (N.W.); cxx18456199339@163.com (X.C.); yangn231026@163.com (N.Y.)

* Correspondence: zhaoying941013@163.com (Y.Z.); xiaoshu2397@163.com (X.Z.); Tel.: +86-244-352-0309 (Y.Z.); +86-244-352-0309 (X.Z.)

† These authors contributed equally to this work and should be considered co-first authors.

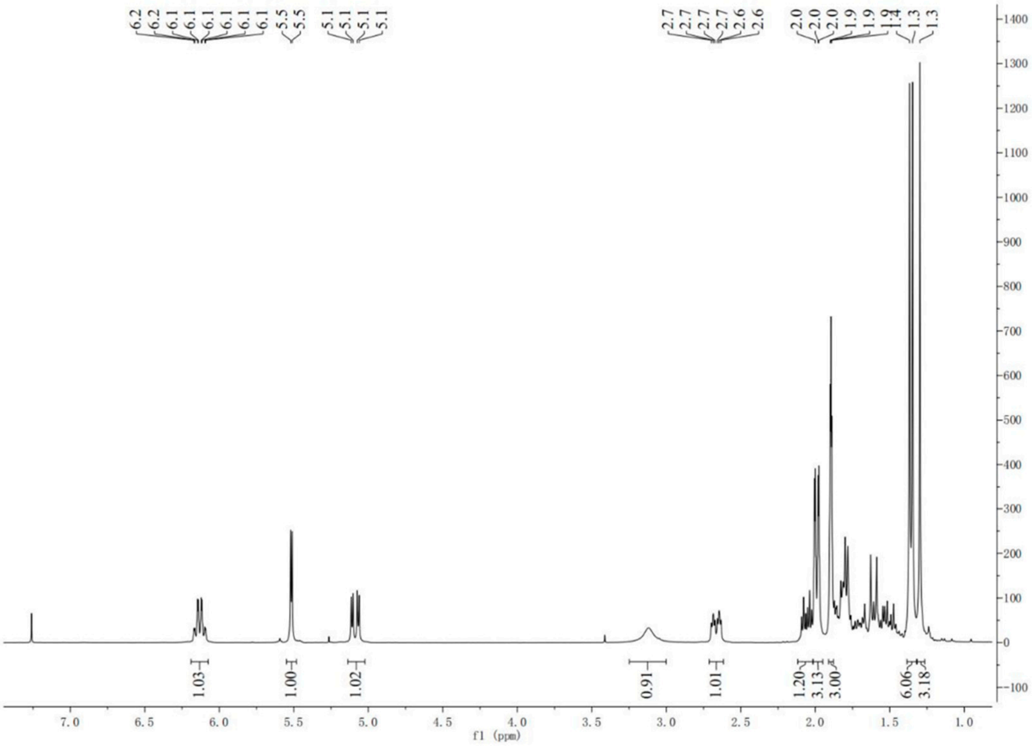

Figure S1. ^1H NMR of CA

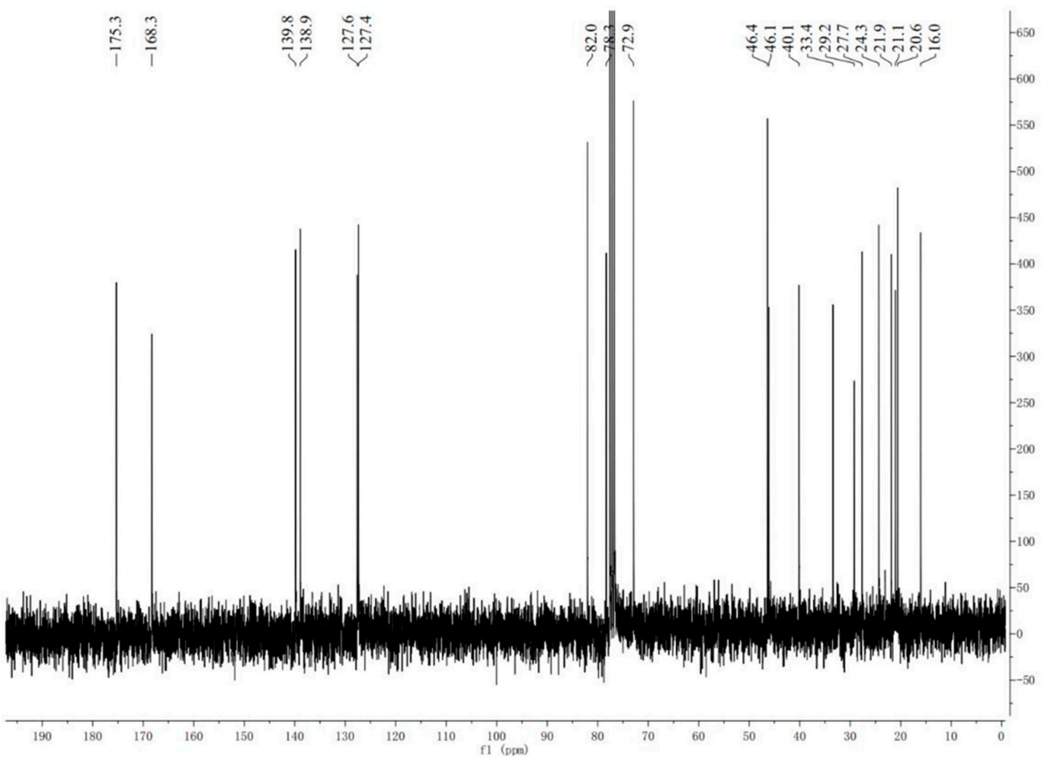

Figure S2. ^{13}C NMR of CA
